# Comparison of the Possibilities of Environmental Usage of Sewage Sludge from Treatment Plants Operating with MBR and SBR Technology

**DOI:** 10.3390/membranes11090722

**Published:** 2021-09-21

**Authors:** Robert Kowalik, Jolanta Latosińska, Monika Metryka-Telka, Rafał Porowski, Jarosław Gawdzik

**Affiliations:** Faculty of Environmental, Geomatic and Energy Engineering, Kielce University of Technology, 25-314 Kielce, Poland; jlatosin@tu.kielce.pl (J.L.); monisia5591@wp.pl (M.M.-T.); rporowski@tu.kielce.pl (R.P.); jgawdzik@tu.kielce.pl (J.G.)

**Keywords:** membrane biological reactors, MBR, heavy metals, sewage sludge, mobility, geo-accumulation index, potential ecological risk index, environmental risk determinant

## Abstract

Sewage sludge from sewage treatment plants has soil-forming and fertilising properties. However, sewage sludge cannot always be used in nature, including agriculture. One of the main reasons is the concentration of heavy metals. Sludge from wastewater treatment plants operating in MBR (membrane biological reactor) and SBR (sequential batch reactor) systems was analysed. Studies comparing the risk analysis of the natural use of sludge from MBR and SBR treatment plants were performed for the first time, due to the fact that more and more MBR plants, which are a BAT technology, are being developed in Poland, displacing the classical SBR plants. MBR technology uses a combination of activated sludge and filtration with microfiltration membranes. Wastewater treated in these reactors meets the highest quality standards, both in terms of physicochemical and microbiological aspects. This paper presents studies on the mobility of heavy metals in sewage sludge carried out using the BCR sequential extraction method. Geo-accumulation index (GAI), potential environmental risk index (ER), risk assessment code (RAC), and environmental risk determinant (ERD) were calculated. Heavy metals dominated the stable fractions in all cases. Furthermore, an increased content of copper and cadmium was observed in the MBR sludge. This fact is favourable in view of the efforts to eliminate heavy metals in the environment.

## 1. Introduction

Sewage sludge is formed as a by-product of processes carried out during the treatment of municipal and industrial wastewater. With the development of civilisation, an increase in the amount of sewage sludge is observed worldwide [1,2,3]. There are many methods to manage the sludge, but the most beneficial is to use it for agricultural or natural purposes, due to the fact that it has high land-forming properties [4]. However, the choice of how a particular sludge can be used is strongly dependent on its properties [5,6,7]. Additionally, the use of sludge is subject to many legal regulations [8,9,10]. The use of sludge for agricultural and environmental purposes is highly dependent on the content of micropollutants, especially heavy metals and parasite eggs [11,12].

The permissible heavy metals content, in terms of the use of sewage sludge in Poland and in the world, is presented in Table 1.

Proper management of sewage sludge is crucial, as otherwise it can result in significant environmental pollution. Heavy metals are extremely toxic to the environment, due to the fact that they enter the elemental cycle in the environment [17]. Heavy metals from soil can migrate to plants, which are a source of food for animals and humans [18,19]. Heavy metals, even in minimal concentrations, can cause negative effects on the functioning of human and animal organisms and, as a result, cause various diseases or even lead to death, so it is very important to limit their accumulation in consumed plants [19]. Another negative aspect is the phenomenon of heavy metals leaching from soils, which may negatively affect the condition of the aquatic environment [18].

The aim of this paper was to analyse the influence of sewage treatment technology on the possibilities of natural use of sewage sludge. Sewage sludge from three wastewater treatment plants operating in a membrane biological reactor (MBR) system and three plants operating in a classical sequencing batch reactor (SBR) system was used. MBRs are currently the best available technology (BAT) for wastewater treatment. This paper presents an analysis of the risk of environmental contamination with heavy metals and the speciation of heavy metals in sludge. A point in the Świętokrzyskie voivodeship has been selected as a target location for sludge management.

## 2. Characteristics of Wastewater Treatment Plants Using MBRs

**Figure 1:**
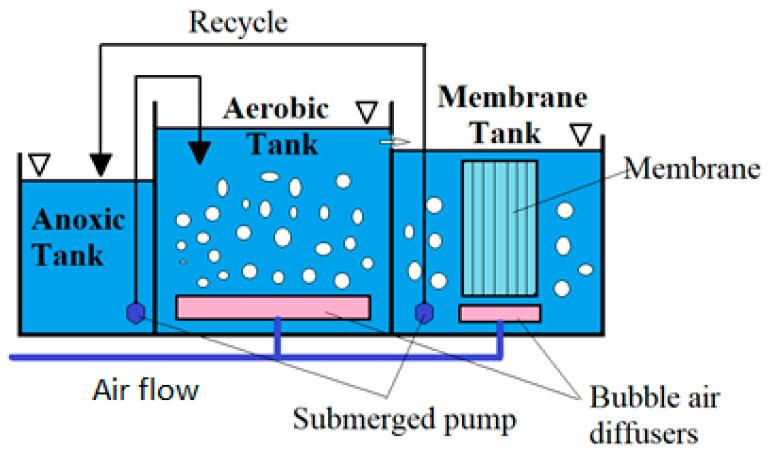
Wastewater treatment in MBRs (membrane biological reactor) (own study).

The modern technique of biological membrane reactors first appeared in the United States of America in the 1970s. This was at the time that ultrafiltration and microfiltration also appeared [20]. Membranes are used in three ways, i.e., for removal of impurities from the solvent, removal of the solvent from the solution, and separation of the solution into components [21]. Figure 1 shows a cross-sectional view and a schematic diagram of the MBR reactor.

MBR technology uses an activated sludge chamber that has been interlocked with a membrane ultrafiltration module, corresponding to a secondary settling tank, and that separates the biomass suspension from the biologically treated effluent, and a biological chamber into which the concentrated biomass is returned [22]. Membrane reactors are an improvement on the activated sludge method. The main difference from the classical activated sludge method is the replacement of the secondary settling tanks by a system of microporous filtration membranes placed directly in the aerated activated sludge chamber or as a device in a separate tank [23]. Membrane biological reactor (MBR) are a combination of pressurised membrane techniques, UF (ultrafiltration) or MF (microfiltration), with a biological treatment method, such as activated sludge. There are two configurations of MBR technology. The first is where the membrane module is immersed in the biological reactor (the membrane is connected to the reactor; in this situation, the activated sludge stays in the reactor and the permeate flows out). In the second case, the membrane module is separated from the reactor (in addition to the permeate, there is a retentate, which is returned to the reactor) [24]. The membrane generally forms a barrier for bacteria, viruses, and protozoa, hence its invaluable use in disinfection of wastewater as well as water [25]. The effect of membrane reactors is the separation of activated sludge from treated wastewater. 

As a result of the impermeability of some compounds through the membranes, activated sludge forms on the membrane, which contributes to the removal of pollutants. Therefore, a decrease in COD and BOD_5_ is observed. According to the literature, removal rates of COD, BOD_5_ (Biochemical Oxygen Demand), TSS (total suspended solids), VSS (volatile suspended solids), and turbidity were 82%, 89%, 98%, 99%, and 98%, respectively [26,27]. On the other hand, as a result of retention of macromolecular compounds by the membrane and longer residence time of activated sludge in the reactor, the possibility of removing pesticides, pharmaceuticals, and hormonal substances (these are micropollutants which are difficult to biodegrade) appears [28]. The structure of membranes in the MBR reactor consists mainly of capillary fibres with a pore diameter of 0.03–0.04 µm. As individual elements, they form bundles which, when immersed in activated sludge, shape the module. Filtration takes place from the outside to the inside of the tube, due to the vacuum created by the filtrate pump. The capillaries float freely in the effluent during the reactor’s operation, which gives continuity of filtration, as no sludge adheres to the outer wall of the membrane. Regular chemical cleaning as well as aeration or backwashing with permeate are automatic procedures to protect the membranes from unnecessary clogging, which could cause poor operation of the entire reactor [29].

### Advantages of Biological Membrane Reactors

Compared to traditional activated sludge reactors, biological membrane reactors have many advantages. The first is that they use membrane filtration (micro or ultra) to separate the treated wastewater from the activated sludge microorganisms, which makes wastewater treatment independent of the sedimentation characteristics of the sludge, i.e., it has a very high separation efficiency and quality of treated wastewater [30]. Another advantage of MBR reactors is the fact that they take up little space, so new sewage treatment plants require smaller construction areas, as well as the possibility of modernising existing plants. The use of MBR reactors also means that most bacteria and viruses are removed, which means that the treated effluent is pre-disinfected and can then be used as process water, as it is free of suspended solids. This technology also makes it possible to bypass the primary settling tank. Due to the high concentration of biomass in the reactor, a shorter retention time of the wastewater is achieved, which results in a three-fold reduction of the volume of the activated sludge chamber. Thanks to the long age of the sludge, we have a more satisfactory process of wastewater treatment as well as reduced generation of excessive sludge and increased throughput, and, without greater costs for the investment area, higher loads of pollutants can be accepted [31]. From a technical point of view, it is worth adding that the replacement of membrane modules is simple, fast, and, above all, cheap. However, it should be taken into account that every technology implemented, besides advantages, also has disadvantages. According to information from wastewater treatment plants with membrane reactors already operating in Poland, various irregularities resulting from their operation have been reported [32]. The most frequently raised issue is the reduction of MBR filtration efficiency under the influence of filtration time. This is due to the deposition of soluble and comminuted materials on and in the membrane, which is caused by interactions between the components of the activated sludge and the membrane. Membranes have a different working range. The suspended biomass does not have a constant composition, and, therefore, it is difficult to determine the general behaviour of a membrane in a MBR [33]. A fouled membrane is an increase in hydraulic resistance associated with a decrease in permeate flux or an increase in transmembrane pressure. This results in an increase in the energy required to achieve the required filtration effect. With all this comes alternative cleaning of the membranes, which significantly increases operating costs [34].

## 3. Materials and Methods

### 3.1. Characteristics of Wastewater Treatment Plants and Sites for Agricultural Use of Sludge

**Figure 2:**
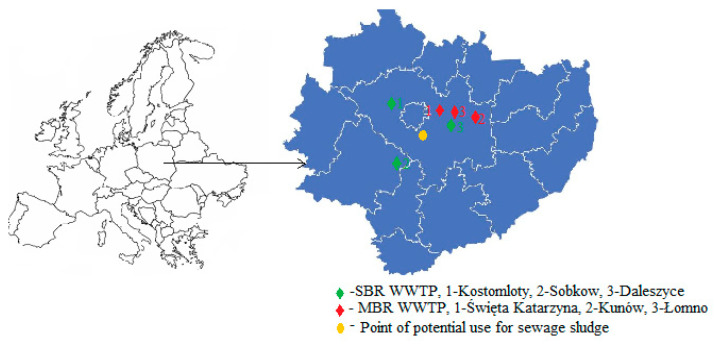
Location of wastewater treatment plants (own research).

Sludge samples were collected from three different wastewater treatment plants located in Poland operating in MBR systems and, for comparison, from three plants in SBR systems with similar p.e. values. The characteristics of the wastewater treatment plants are presented in Table 2. The Masłów measurement point developed within the framework of the Monitoring of Soil Chemistry in Poland [35], located in close proximity to the sewage sludge sites, was used as a comparative point for heavy metal contents in soil (Figure 2).

### 3.2. Sewage Sludge Analysis

Analyses of organic matter and dry matter content were conducted in accordance with PN-EN 12880:2004 and PN-EN 12879:2004 standards. For pH and redox potential (Eh) measurements, a CPR-411 multifunction meter (Elmetron) was used. An ICP-OES Perkin Elmer Optima 8000 inductively coupled plasma optical emission spectrometer (Waltham, MA, USA) was used to determine heavy metal content. Tests were performed on sludge samples taken immediately after the treatment processes, after which the sludge was fully dried to dry weight. Four equivalent tests were performed for each sludge to exclude coarse errors. Statistical analysis of the results was performed in MS Excel.

### 3.3. Mobility of Heavy Metals

Depending on the migration capacity of metals into the soil environment, they can be assigned to four fractions [36]. The first fraction, the most mobile FI, is associated with carbonates; the second fraction, FII, is associated with amorphous iron and manganese oxides; the third fraction, FIII, conditionally mobile, is associated with organic matter; while the fourth fraction, FIV, the most stable—practically not penetrating into soils—is associated with silicates. The BCR sequential extraction procedure [37] was used to analyse the metal content of the different fractions and is shown in Table 3.

### 3.4. Risk Indicators for Accumulation of Heavy Metals

#### 3.4.1. Geo-accumulation Index of Heavy Metals in Soil (Igeo)

One of the indicators for assessing heavy metal accumulation in soils is the Igeo index, which uses the metal content of sewage sludge and soil and is described by the equation [40,41]:

(1) $$\mathrm{Igeo}=\log_{2}\frac{C_{n}}{1.5\cdot B_{n}}$$

where:

*C_n_*—Heavy metal content of sewage sludge, mg/kg d.m.;
*B_n_*—Heavy metal content in soil, mg/kg d.m.

Table 4 presents the classification of the heavy metal geo-accumulation index.

#### 3.4.2. Risk Assessment Code (RAC)

Another indicator used to assess the risk of environmental contamination with heavy metals is the risk assessment code (RAC) indicator [45]. The RAC is an indicator that takes into account the mobility of heavy metals. It analyses the contribution of the FI fraction, the most mobile fraction, to the total content of a given heavy metal [45,46]. The risk level is classified into five groups, which are presented in Table 5, while the indicator itself is defined by the following formula [45]:

(2) $$\mathrm{RAC}=\frac{F1}{\mathrm{HM}}\cdot 100\%$$

where:

F1—heavy metal content of the FI fraction, mg/kg d.m.;
HM—total heavy metal content, mg/kg d.m.

**Table 5:** Classification of RAC [45].

RAC Value	Level of Pollution
<1	No pollution
1–10	Low pollution
11–30	Moderate pollution
31–50	High pollution
>50	Very high pollution

#### 3.4.3. Potential Environmental Risk Indicator (ER)

The indicator of potential environmental risk (ER) is the third indicator analysed. It takes into account the heavy metal content of soils, as for Igeo [40,42]. However, each metal is assigned a different toxicity level, which is defined as a toxicity factor—for Zn, 1; Cr, 2; Pb, Cu, and Ni, 5; and 30 for Cd [46]. ER is described in the following equations [40,42]:

(3) $$C_{f\;}^{i}=\frac{C_{D}^{i}}{C_{R}^{i}}$$

where:

$C_{f}^{i}$—pollution factor;
$C_{D}^{i}$—concentration of the *i*-th element of heavy metals in sewage sludge, mg/kg d.m.;
$C_{R}^{i}$—concentration of the *i*-th element of heavy metals in soil, mg/kg d.m.

(4) $$E_{r}^{i}=T_{r}^{i}\cdot C_{f}^{i}$$

where:

$E_{r}^{i}$—indicator of the potential ecological risk of the *i*-th element of heavy metals;
$T_{r}^{i}$—toxicity factor of the *i*-th element of heavy metals.

The risk level of environmental contamination according to the ER indicator is shown in Table 6.

#### 3.4.4. Environmental Risk Determinant (ERD)

The fourth indicator analysed is the environmental risk determinant (ERD). It uses the mobility of heavy metals, but, unlike the RAC indicator, it additionally takes into account the FII and FIII fractions, which are conditionally mobile [47]. The FIII fraction, considered to be more stabilised, may become mobile under specific conditions, such as when soil ozone is high after a storm [47]. It, therefore, makes sense to analyse all three fractions to check the actual risk of environmental contamination. However, given that the FI fraction is the most mobile and the FIII fraction the least, appropriate value weights for each fraction should be applied. Each fraction is assigned an appropriate value scale of 0–1. The ERD index is described by the equation [18]:

ERD = F_p1_ + F_p2_ + F_p3_ (5)

where:

F_p1_ = F_1_; F_1_—metal content in fraction FI on a scale of 0–1;
F_p2_ = F_2_^2^; F_2_—metal content in fraction FII on a scale of 0–1;
F_p3_ = F_3_^3^; F_3_—metal content in fraction FIII on a scale of 0–1.

The classification of the ERD results is: 0 < ERD ≤ 0.35, low risk; 0.35 < ERD ≤ 0.6, medium risk; 0.6 < ERD ≤ 0.8, high risk; and 0.8 < ERD, very high risk.

## 4. Results and Discussion

Table 7 presents the results of the speciation analysis of heavy metals in sewage sludge. Sewage sludge from wastewater treatment plants mostly met the requirements of acceptable heavy metal content for agricultural purposes according to the Regulation of the Minister of Environment of 6 February 2015 [9] and EU Directive 86/278/EEC [13]; the exceptions were cadmium for WWTP1 and zinc for WWTP6. Sewage sludge collected from wastewater treatment plants operating in MBR systems was characterised by an increased content of copper and cadmium, compared to sludge from SBR treatment plants. The highest heavy metal contents were observed in the most stable fraction FIV and the conditionally mobile fraction FIII. The metal contents in the mobile fractions were negligible, except for chromium and cadmium for WWTP4, which dominated in the FI fraction. According to literature data, concentrations of individual heavy metals in sewage sludge can be ranked as follows: Zn > Cu > Cr > Ni > Pb > Cd [48]. The study showed that the trend in metal concentrations was as follows: Zn > Cu > Cr > Pb > Ni > Cd for WWTP1; Zn > Cu > Ni > Pb > Cr > Cd for WWTP2; Zn > Cu > Ni > Cr > Pb > Cd for WWTP3; Zn > Pb > Cr > Cu > Cd > Ni for WWTP4; Zn > Cu > Pb > Cr > Ni > Cd for WWTP5; and Zn > Pb > Cr > Cu > Ni > Cd for WWTP6. As can be seen, the results differed slightly between each other as well as from the literature data. 

**Figure 3:**
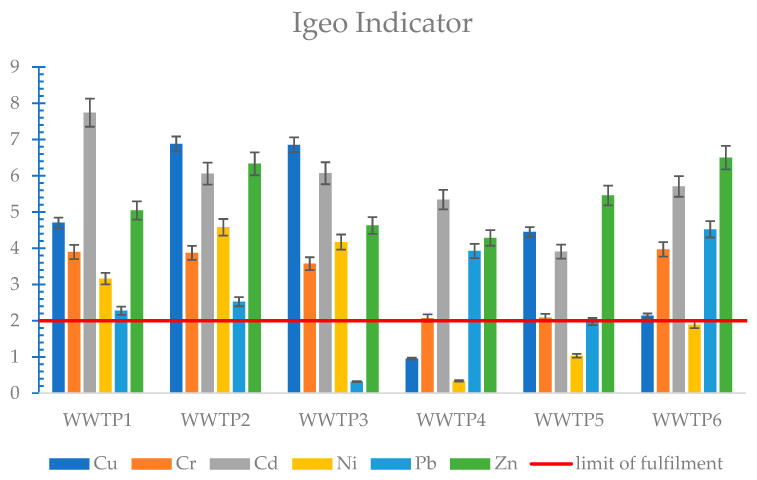
Igeo indicator of heavy metals in sewage sludge.

**Figure 4:**
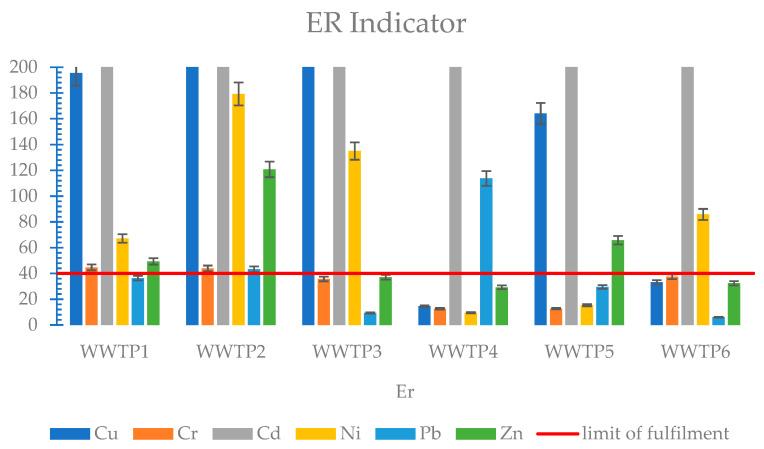
ER indicator of heavy metals in sewage sludge.

Analysing the levels of the heavy metal geo-accumulation index (Igeo) in the soil, it can be concluded that the risk level of heavy metal contamination in the environment is very high. All metals coming from sludge from membrane treatment plants (S1–S3), with the exception of lead, showed a high risk of contamination (Figure 3). Sludge from treatment plants operating with SBR technology also showed high Igeo values; only nickel was at a lower level compared to sludge from MBR plants (Figure 4). The metals that caused the highest contamination risk for all sludges were cadmium, copper, and zinc. The Igeo index compares the metal content of sewage sludge to that of soil. The value of this indicator is, therefore, strongly dependent on the quality and condition of the soil at the site of potential use.

The ER indicator was also very stringent for the analysed sludge. Cadmium posed a very high risk of ecological contamination for sludge from all six WWTPs. Copper also showed very high risk values for sludge from all three membrane treatment plants as well as WWTP5. Another heavy metal of high risk was nickel for WWTP2, WWTP3, and WWTP6. The remaining metals posed a low to moderate risk, with the exception of zinc for WWTP2 and lead for WWTP4 (Figure 4).

**Figure 5:**
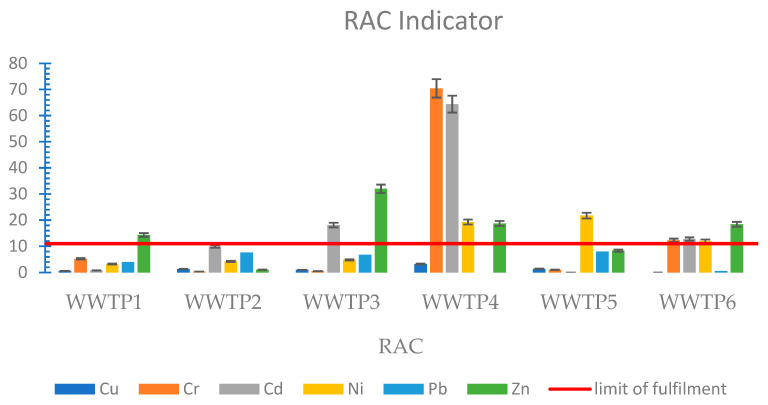
RAC indicator of heavy metals in sewage sludge.

The risk assessment code uses the concept of heavy metal mobility. It takes into account the ratio of the metal content of the first fraction (the most mobile fraction, which tends to migrate deep into the soil) to the total metal concentration. In most cases, the level of the RAC index did not show a high ecological risk. This was due to the low proportion of heavy metals in the most mobile fraction (FI). The exceptions were chromium (70.42% FI) and cadmium (64.38% FI) for WWTP4 and zinc (31.99% FI) for WWTP3 (Figure 5).

**Figure 6:**
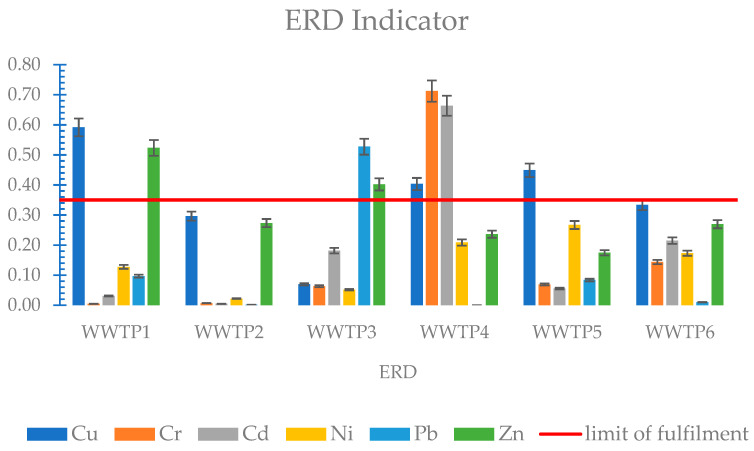
ERD indicator of heavy metals in sewage sludge.

Analysing the results of the ERD indicator, it can be concluded that most of the metals show a low risk level (Figure 6). Only chromium and cadmium for WWTP4 showed a high risk, as for the RAC indicator. The ERD, compared to the RAC indicator, showed a significantly higher risk for copper for most of the sediments and also for lead for WWTP3. This difference is due to the fact that the RAC index does not consider the second and third mobility fractions to any extent.

For the results of heavy metal toxicity according to the analysed indicators, non-compliance tables were created for each wastewater treatment plant. These tables included those heavy metals that did not meet the criterion of being classified as having no negative environmental impact and causing no pollution. This is shown in Table 8. The Igeo and ER indices proved to be the most stringent, which is due to the fact that these two indices take into account the heavy metal content at the site of potential use, while they do not take into account the issue of heavy metal mobility.

## 5. Conclusions

The analysed sewage sludge, with the exception of cadmium for WWTP1 and zinc for WWTP6, meets the applicable limits for heavy metal content, which is one of the basic criteria determining the possibility of using it for natural purposes, including agriculture. Cadmium for WWTP1 reached 38.46 mg/kg and exceeded by 92% the permissible concentration dictated by the law, while zinc for WWTP6 reached 2769.4 mg/kg, exceeding the limits by 11%. The Igeo and ER hazard indices proved to be very stringent for the analysed sewage sludge. The highest Igeo and ER values were found for cadmium at WWTP6, which was 7.74 for Igeo and 9615 for ER, respectively. Nickel, on the other hand, did not exceed the permissible Igeo value for all sludges from the SBR plant. According to the ER index, chromium was found to be the least toxic heavy metal for all treatment plants. The RAC indicator, for the most part, showed a low risk of environmental contamination, except for chromium and cadmium for WWTP4, which were 70.42% and 64.38%, respectively. According to the ERD indicator, as in the case of the RAC indicator, chromium (0.71) and cadmium (0.66) for WWTP4 showed the highest risk, with, additionally, high values for copper (0.59) and zinc (0.52) for WWTP1 and lead (0.53) for WWTP3.

Despite the fact that the sludge fulfilled the conditions with regard to heavy metal content dictated by the legislation, it carried a high risk for natural use, including agricultural. In the case of the analysed sludge from treatment plants with low p.e. values, it would be more advisable to use the sludge in an alternative way, e.g., in the production of lightweight aggregates.

Significantly higher copper and cadmium contents were observed for sludge collected from MBR plants compared to sludge from SBR plants. Therefore, it can be concluded that the membranes built into the process line of the plant absorb increased amounts of heavy metals (mainly cadmium and copper) from the treated wastewater. Thanks to more effective retention of heavy metals in sewage sludge through the use of membranes, a smaller portion of them reaches the receiving body together with the treated wastewater. In order to minimise the impact of human activity on the environment, it would be advisable to introduce membranes in already operational treatment plants.

## Figures and Tables

**Table 1:** Normative limit values for heavy metals in sewage sludge for natural use.

Region	Heavy Metals [mg/kg d.m.]					
	Cd	Ni	Zn	Cu	Cr	Pb
Polish Regulation [9]	20	300	2500	1000	500	750
EU Directive 86/278/EEC [13]	20–40	300–400	2500–4000	1000–1750	-	750–1200
Chinese Regulation GB 18918-2002 pH > 6.5 [14]	20	200	1000	500	1000	1000
USA Regulation 40 CFR Part 503, 503.13 [15]	39	420	2800	1500	-	300
South African Guideline (Pollutant Class a) [16]	40	42	2800	1500	1200	300

**Table 2:** Characteristics of WWTPs.

Type of WWTP	Membrane Biological Reactor (MBR)			Sequential Batch Reactor (SBR)		
Location of WWTP	S1-Święta Katarzyna	S2-Kunów	S3-Łomno	S4-Kostomłoty-Laskowa	S5-Sobków	S6-Daleszyce
Equivalent Number of Residents	2605	6687	3863	3333	3725	5000

**Table 3:** Method of metal speciation of heavy metals in sewage sludge [38,39].

Fraction	Form of Metal	Parameters of Fractionation	Time of Extraction, h
FI	Carbonate bound	0.11 M CH_3_COOH, pH = 7.0, T = 20 °C	16
FII	Fe/Mn oxides bound	0.1 M NH_2_OH·HCl, pH = 2.0	16
FIII	Organic	30% H_2_O_2_ + 8.8 M H_2_O_2_, pH = 2.0, T = 85 °C	16
FIV	Residual	10 M HNO_3_ + 10 M HCl, T = 100 °C	3

**Table 4:** Classification of Igeo [41,42,43,44].

Igeo Value	Level of Risk
<0	No pollution
0–1	No pollution, moderate pollution
1–2	Moderate pollution
2–3	Moderate or high pollution
3–4	High pollution

**Table 6:** ER indicator classification [40,41,42].

$E_{r}^{i}$ Value	Level of Risk
<40	Low
40–80	Medium
80–320	High
>320	Very high

**Table 7:** Chemical speciation of heavy metals in sewage sludge, mg/kg d.m. (Heavy metal content with standard deviation calculated for 4 samples using Grubbs‘ statistical tests).

Heavy Metal [mg/kg d.m.]						
Fraction	Cu	Cr	Cd	Ni	Pb	Zn
MBR WWTP Sewage sludge						
Sewage sludge Święta Katarzyna—S1						
Fraction I	7.53 ± 0.1	0.37 ± 0.1	1.19 ± 0.1	4.59 ± 0.2	7.97 ± 0.2	26.2 ± 1.2
Fraction II	0.44 ± 0.1	0.21 ± 0.1	0.24 ± 0.1	0.31 ± 0.5	0.83 ± 0.1	8.45 ± 0.8
Fraction III	101.92 ± 0.9	17.8 ± 0.9	3.48 ± 0.1	10.35 ± 0.1	17.05 ± 0.3	795.87 ± 9.8
Fraction IV	15.14 ± 0.1	82.18 ± 2.8	33.55 ± 0.2	25.03 ± 0.3	62.15 ± 0.3	176.14 ± 2.0
ΣFI IV	125.03 ± 0.9	100.56 ± 2.9	38.46 ± 0.2	40.28 ± 0.6	88.00 ± 0.05	1006.66 ± 10.1
Sewage sludge Kunów—S2						
Fraction I	2.72 ± 0.1	0.16 ± 0.1	0.07 ± 0.1	0.76 ± 0.5	0.4 ± 0.1	53.93 ± 1.1
Fraction II	7.39 ± 0.6	0.09 ± 0.1	0.00 ± 0.1	0.54 ± 0.5	0.00 ± 0.2	29.13 ± 1.2
Fraction III	298.64 ± 0.9	18.89 ± 0.9	2.04 ± 0.2	30.64 ± 0.1	12.57 ± 0.2	1544.97 ± 15
Fraction IV	255.62 ± 0.2	79.71 ± 2.7	9.88 ± 0.1	75.60 ± 0.1	91.60 ± 1.3	835.44 ± 4.2
ΣFI IV	564.36 ± 0.4	98.84 ± 2.8	11.99 ± 0.2	107.53 ± 0.7	104.57 ± 0.3	2463.46 ± 15.6
Sewage sludge Łomno—S3						
Fraction I	5.49 ± 0.1	0.44 ± 0.1	2.19 ± 0.1	3.91 ± 0.2	1.52 ± 0.1	242.04 ± 3.3
Fraction II	14.38 ± 0.1	0.13 ± 0.1	0.28 ± 0.1	1.85 ± 0.1	4.01 ± 0.2	110.38 ± 9.1
Fraction III	225.79 ± 0.6	17.88 ± 0.9	1.35 ± 0.4	9.04 ± 0.6	17.1 ± 0.1	296.01 ± 7.1
Fraction IV	309.45 ± 0.1	61.85 ± 2.4	8.26 ± 0.1	66.22 ± 0.4	253.52 ± 9.3	108.22 ± 9.1
ΣFI IV	555.11 ± 0.6	80.30 ± 2.6	12.08 ± 0.4	81.02 ± 0.6	22.65 ± 9.3	756.65 ± 15.1
SBR WWTP Sewage sludge						
Sewage sludge Kostomłoty—S4						
Fraction I	0.3 ± 0.1	20 ± 0.9	4.7 ± 0.4	1.1 ± 0.1	0.0 ± 0.2	111.5 ± 9.3
Fraction II	0.0 ± 0.1	3.2 ± 0.2	0.9 ± 0.1	0.3 ± 0.5	0.0 ± 0.2	109.7 ± 9.1
Fraction III	6.7 ± 0.6	1.5 ± 0.1	1.5 ± 0.2	1.4 ± 0.2	0.0 ± 0.2	143.4 ± 9.8
Fraction IV	2.3 ± 0.1	3.7 ± 0.4	0.3 ± 0.1	2.9 ± 0.2	275.2 ± 9.5	231.5 ± 7.1
ΣFI IV	9.3 ± 0.6	28.4 ± 1.0	7.3 ± 0.5	5.7 ± 0.6	275.2 ± 9.5	596.0 ± 17.8
Sewage sludge Sobków—S5						
Fraction I	1.5 ± 0.1	0.3 ± 0.1	0.0 ± 0.1	2.0 ± 0.3	5.7 ± 0.5	111.6 ± 2.0
Fraction II	1.0 ± 0.1	0.0 ± 0.1	0.2 ± 0.1	1.4 ± 0.1	4.6 ± 0.4	215.2 ± 3.3
Fraction III	79.5 ± 0.3	11.2 ± 0.1	1.0 ± 0.1	2.7 ± 0.1	4.3 ± 0.5	556.6 ± 4.2
Fraction IV	23.0 ± 0.2	17.1 ± 0.5	1.5 ± 0.1	3.1 ± 0.2	49.8 ± 0.7	457.9 ± 4.1
ΣFI IV	105.0 ± 0.4	28.6 ± 0.5	2.7 ± 0.2	9.2 ± 0.4	71.4 ± 1.1	1341.3 ± 7.0
Sewage sludge Daleszyce—S6						
Fraction I	0.0 ± 0.1	13.0 ± 0.9	1.2 ± 0.1	2.0 ± 0.2	2.5 ± 0.2	509.9 ± 9.0
Fraction II	0.0 ± 0.1	4.2 ± 0.2	1.5 ± 0.1	0.9 ± 0.1	0.0 ± 0.1	447.3 ± 9.5
Fraction III	14.6 ± 0.9	29.4 ± 1.6	3.7 ± 0.1	6.1 ± 0.5	16.2 ± 0.3	1119 ± 15
Fraction IV	6.5 ± 0.4	59.1 ± 2.3	3.0 ± 0.1	7.7 ± 0.6	408.4 ± 9.1	693.2 ± 8.4
ΣFI IV	21.1 ± 0.9	105.7 ± 2.9	9.4 ± 0.2	16.7 ± 0.8	417.1 ± 3.8	2769.4 ± 21,6

**Table 8:** Table of non-compliance with heavy metal toxicity criterion from analysed sites by pollutant indicators.

Indicator	S1	S2	S3	S4	S5	S6
Igeo	Cu, Cr, Cd, Ni, Pb, Zn	Cu, Cr, Cd, Ni, Pb, Zn	Cu, Cr, Cd, Ni, Zn	Cr, Cd, Pb, Zn	Cu, Cr, Cd, Zn	Cu, Cr, Cd, Ni, Pb, Zn
ER	Cu, Cr, Cd, Ni, Zn	Cu, Cr, Cd, Ni, Pb, Zn	Cu, Cd, Ni	Cd, Pb	Cu, Cd, Zn	Cd, Ni
RAC	Zn	-	Cd, Zn	Cr, Cd, Ni, Zn	Ni	Cr, Cd, Ni, Zn
ERD	Pb, Zn	-	Ni, Zn	Pb, Cd, Cr	Pb	-

## Data Availability

The datasets supporting the results of this article are included within the article and its additional files.
